# Genetic Testing as a Source of Information Driving Diagnosis and Therapeutic Plan in a Multidisciplinary Case

**DOI:** 10.3390/bioengineering11101023

**Published:** 2024-10-14

**Authors:** Cristina Grippaudo, Concetta Cafiero, Nicola Maria Grande, Leonardo Dassatti, Raffaele Palmirotta, Raffaella Castagnola, Gaetano Isola

**Affiliations:** 1 UOC di Clinica Odontoiatrica, Dipartimento di Neuroscienze, Organi di Senso e Torace, Fondazione Policlinico Universitario A. Gemelli, IRCCS, 00168 Rome, Italy; cristina.grippaudo@unicatt.it; 2 Dipartimento Universitario Testa Collo ed Organi di Senso, Università Cattolica del Sacro Cuore, 00168 Rome, Italy; nicolamaria.grande@unicatt.it (N.M.G.); leonardo.dassatti2@unicatt.it (L.D.); 3 Medical Oncology, SG Moscati Hospital, 74010 Taranto, Italy; concettacafiero@gmail.com; 4 Interdisciplinary Department of Medicine, University of Bari “Aldo Moro”, 70124 Bari, Italy; raffaele.palmirotta@uniba.it; 5 Department of General Surgery and Surgical-Medical Specialties, School of Dentistry, University of Catania, 95123 Catania, Italy; gaetano.isola@unict.it

**Keywords:** genes, orthodontic appliances, tooth resorption

## Abstract

In many cases, the etiopathogenesis of oral cavity diseases depends on the presence of variants in some genes. Being able to identify these variants defines the possibilities and limits of therapies. This multidisciplinary case describes several pathologies of the oral cavity in a young patient affected by type 1 diabetes. The patient presented with an impacted palatal canine. Further investigation revealed cervical root resorption of the upper right central incisor. Genetic testing was performed for interleukin, VDR receptor genes, and the evaluation of periodontopathogenic bacteria. The mutational analysis carried out for the VDR polymorphisms and the IL1A, IL1B, IL6, and IL10 polymorphisms showed the presence of pathogenetic variants. The results for bacterial load showed the presence of periodontal pathogenes. The first intervention was the intentional replantation of the incisor. The second intervention was the orthodontic recovery of the impacted canine, using light forces and a hybrid anchorage with a miniscrew. At the end of orthodontic treatment, a crack was found in the upper left first premolar, which was extracted. Throughout treatment, non-invasive periodontal interventions were performed periodically to control periodontal inflammation. This case is an example of the integration of genetic analyses into the multidisciplinary diagnostic pathway.

## 1. Introduction

In dentistry, a multidisciplinary clinical case is characterized by the presence of several problems that require the coordinated intervention of a team of specialists from different branches of dentistry. Diagnostic evaluations must consider the patient’s state of health, the treatments to which he or she is subjected, and the possible causal factors of the dental pathologies observed. The progress of research in dentistry has allowed us to understand the role of genetic factors in the pathogenesis of some oral diseases [1]. It is therefore hypothesized to use a personalized approach to set the therapeutic plan based on the specific characteristics of the individual patient. Consequently, we speak of personalized medicine when diagnostic tests, often genetic, allow us to identify the causes underlying the presence of clinical signs. Response to therapies can be predicted based on genomics, proteomics and metabolomics. Furthermore, numerous studies have made it possible to study the microbiota and microbiome of the bacteria present in the oral cavity, clarifying their role in the most widespread dental diseases [2]. However, current knowledge is still incomplete, as it is possible to observe many phenotypes carrying the same gene variant. Furthermore, we do not yet have sufficient knowledge to precisely understand the biological response to dental therapies [3]. However, the information derived from genetic tests can help assess risks and guide the clinician in planning interventions and monitoring expected effects to avoid adverse reactions.

Periodontitis is a multifactorial disease, whose staging and grading criteria were defined in 1999 and modified in 2017 [4]. Among the risk factors, the presence of pathogenic bacteria is recognized, whose proliferation can be favored by the patient’s genetics. Diabetes is indicated as an aggravating factor of the risk of worsening over time. The cluster called “red complex” are bacterial species associated with severe periodontal disease [5]. Furthermore, the role of other bacterial phyla is known among the causes of periodontitis [6]. Many studies have investigated the role of multiple cytokine gene polymorphisms in determining periodontitis [7]. Vitamin D receptor (VDR) polymorphisms appear to be related to the presence and level of periodontal pathogens, qualifying the genetic predisposition to develop periodontal disease [8,9]. In this regard, it has been reported that individuals with periodontitis often exhibit changes in both their oral composition and diversity [10]. Moreover, across various consortia, the causal relationships between oral and gut microbiome taxa and periodontitis exhibit significant variability, suggesting that specific oral and gut taxa may serve as novel biomarkers or therapeutic targets for periodontitis [11].

Inclusions are dental problems often caused by genetic alterations. The affected teeth fail to erupt into the dental arch and remain inside the jaw. Canines are among the most frequently affected teeth due to inclusion, with the possibility of assuming a more or less angulated position compared to the normal eruptive path, with a higher frequency in the upper jaw and in a palatal position. The probability that the defect is due to a genetic alteration is greater if the deciduous canine is preserved in the arch and the space available for the exchange is adequate [12]. Restoring an impacted canine exposes the patient to the risk of failure and complications, which increase with age. One of the risks is the possibility of developing ankylosis and invasive cervical root resorption (ICRR), which is a rare case of external cervical resorption (ECR) [13]. The cause of the onset of ICRR is not yet clear, but it seems to depend on the method chosen when performing the surgery required to hook the impacted canine, the traction method, and genetic alterations of the inflammatory mediators in the periodontal ligament. The search for genetic factors responsible for tooth resorption has led to the identification of some pathogenic variants [14]. Variants of some interleukins appear to play a role in external apical root resorption (EARR) [15]. External apical root resorption (EARR) may have an iatrogenic cause, because it can be caused by the application of excessive orthodontic force in a genetically predisposed patient [16]. The amount of orthodontic force applied depends on the distribution of the anchorage on the teeth that must serve as support for the active orthodontic mechanism for treatment. It also depends on the choice of appliance, orthodontic wires and the technique used. Temporary Anchorage Devices (TADs) with miniscrews can be used to reduce the anchorage load on the teeth. Many authors have proposed integrated orthodontic systems with the use of TADS for the recovery of impacted canines. The success and failure of miniscrew therapy depends on the oral microbiome [17,18]. Teeth that have erupted normally in the arch but that undergo ECR are treated by an endodontist, and it is sometimes necessary to perform an intentional replantation. Intentional replantation is a technique that involves the voluntary extraction of the tooth, treatment of the root, and its repositioning in the original socket [19,20]. This clinical procedure can be used to manage different situations, including ECR [20], and some authors have reported a survival rate of approximately 90%, showing that it can be a good, reliable, and cost-effective treatment option [21,22]. The case report is described as an example of multidisciplinary treatment, and it presents many pathological aspects dependent on the alteration of genetic factors. The case report focuses on a diabetic patient, with a palatally impacted canine and with the external cervical resorption of an upper central incisor. In this report, genetic analyses to identify the oral microbiome and interleukin variants were essential to this patient’s diagnosis and treatment plan, which was discussed as part of multidisciplinary management. The therapeutic choices therefore took into account the risk factors inherent in the patient’s genetics, with the aim of personalizing care.

## 2. Case Report

**Figure 1:**
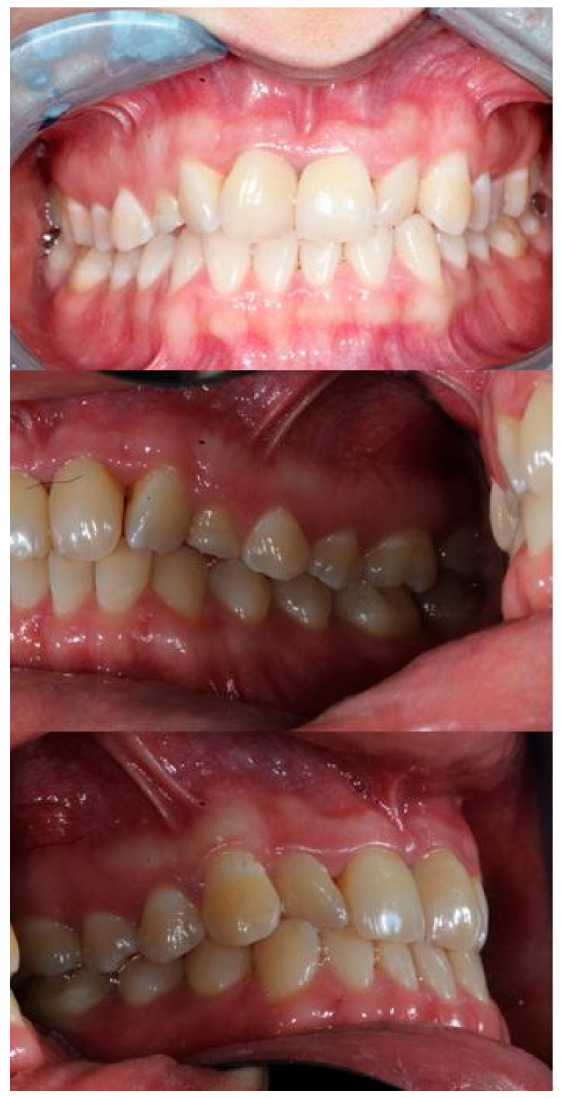
Intraoral photograph of the patient in 2018 at the beginning of treatment.

In January 2018, a Caucasic 22-year-old woman first visited an orthodontist at the Division of Oral Surgery and Implantology at the Università Cattolica del Sacro Cuore, Fondazione Policlinico Gemelli IRCCS, Rome, Italy. The reason for the visit was the lack of the upper right canine in the arch and the permanence of the deciduous canine. As anamnestic data, she reported that she was suffering from type 1 diabetes and had never undergone dental treatment. The patient presented a malocclusion characterized by crowding of the upper and lower incisors, crossbite of the left lateral incisor, and absence of the upper right canine (Figure 1).

Regarding periodontal status, no presence of periodontal diseases was reported, with a mean probing pocket depth (PPD) < 4 mm and a mean bleeding on probing (BoP) < 10%. The analysis of the gingival tissues also evidenced the presence of a right band of keratinized tissues and a thick gingival biotype.

**Figure 2:**
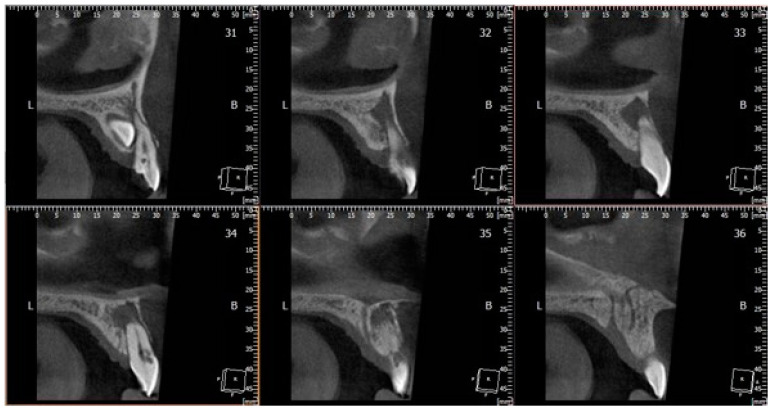
This image shows a sagittal view of 1.1 and 1.3 teeth on the cone beam computed tomography (CBCT) scan. ECR, asymptomatic apical periodontitis, and the impacted canine are visible.

A cone beam computed tomography (CBCT) scan of the dental arches was requested to evaluate the position of the impacted canine. The radiographic examination showed the presence of the impacted canine in a palatal position, also allowing the cervical resorption of the root of the right upper central incisor to be revealed and assessed to be ECR Class 3 [23] (Figure 2).

**Figure 3:**
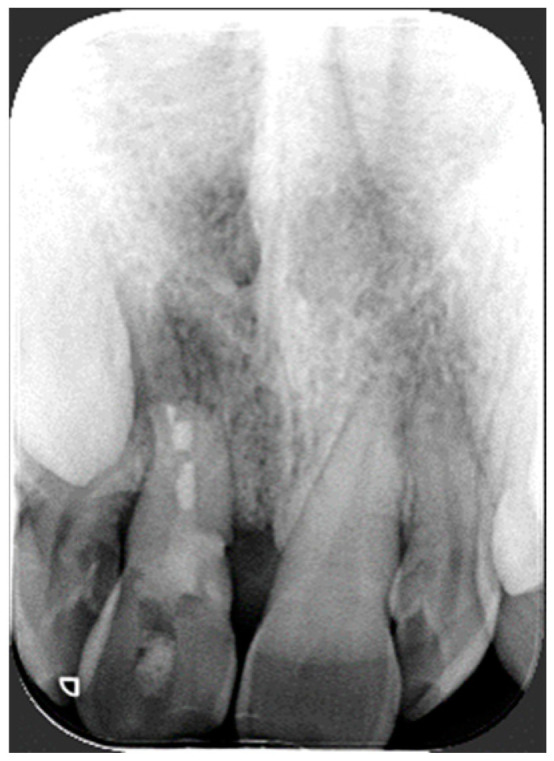
Periapical radiography of the 1.1 tooth after intentional reimplantation and the removal of the splint.

It was hypothesized that a possible genetic variation could favor the damage to the integrity of the incisor as for external root resorption and multiple idiopathic cervical root resorption [24,25,26,27] and in this case, interleukin and VDR receptor genes were evaluated. Therefore, DNA was collected using an oral brush. At the same time, a series of biological samples were collected from the deepest periodontal pocket in each quadrant of the dentition using sterile paper points to identify and quantify periodontal pathogens, and the sample was sent to the laboratory. Written informed consent, including information about the diagnostic procedures and genetic testing, was obtained from the patient before her participation in the study and biological sampling. Awaiting the results of molecular testing, the endodontist took care of the patient, who treated the central incisor with cervical resorption. Subsequently, the patient returned to the orthodontist to plan and implement the disinclusion of the palatal canine. Regarding the cervical resorption of 1.1, the surgical approach was excluded due to the location of the defect, and consequently, an intentional reimplantation was performed [20]. After anesthesia, extraction forceps were used to gently extract the incisor. Under the microscope, holding the tooth with a gauge, all the granulation tissue was removed with a diamond bur, and the defect was filled with a composite material. Then, a retro-preparation until ECR was carried out with a diamond bur and ultrasonic endodontic tip specific for surgical endodontics and filled with MTA. A socket curettage was completed, rinsed with saline solution, and then, the tooth was reimplanted and held under finger pressure before being stabilized with a flexible splint for 2 weeks [28] (Figure 3).

### 2.1. Identification and Quantification of Periodontal Pathogens

Biological samples useful for the evaluation of periodontopathogenic bacteria were obtained by inserting sterile paper tips into the deepest periodontal pocket for 30 s and then placing them in sterile 1.5 mL tubes containing 300 μL of sterile phosphate buffer [9,29]. These samples were then stored in a freezer at −80 °C until DNA extraction using the Ampli DNA EXTRA kit (Dia-Chem Srl, Molecular Biology, Naples, Italy) according to the manufacturer’s protocol. DNA concentration was measured using a NanoDrop device (Thermo Fisher Scientific, Waltham, MA, USA), and samples with a ratio of A260/280 ≥ 1.8 were considered suitable for further analysis [30]. Specific periodontopathogenic bacterial species were identified and quantified as previously described [9]. In brief, species-specific primers and probes designed from variable regions of the 16S rRNA gene for *Aggregatibacter actinomycetemcomitans, Porphyromonas gingivalis, Porphyromonas endodontalis, Treponema denticola, Tannerella forsythia*, and *Prevotella intermedia* were used to perform real-time PCR in a cycler CFX96 Touch Real-Time PCR Detection System (Biorad, Boston Industries, Inc., Walpole, MA, USA). The results of the quantitative analysis are expressed in copies/mL.

### 2.2. Determination of Genomic Polymorphisms

Patient genomic DNA was obtained using cytobrushes (Cooper Surgical, Trumbull, CT, USA) and extracted using an Ampli DNA EXTRA kit (Dia-Chem Srl, Molecular Biology, Naples, Italy) according to the manufacturer’s protocol [9]. The FokI (rs2228570), BsmI (rs1544410), ApaI (rs7975232), and TaqI (rs731236) VDR polymorphisms were analyzed using the commercial kit AMPLI set VDR Polymorphisms (Dia-Chem Srl, Molecular Biology, Naples, Italy) according to the manufacturer’s procedures. This system consists of initial PCR amplification with specific primers for the gene regions containing the polymorphisms, followed by the use of specific DNA restriction enzymes. Primer pairs for PCR and sequence analysis for the IL1A (rs1800587), IL1B (rs1143634), IL-6 (rs1800795), and IL10 (rs1800896 and rs1800871) polymorphisms were designed using Genamics Expression DNA Sequence Analysis Software 1.7 (Genamics, Hamilton, New Zealand) and the in Silico-PCR tool provided by the UCSC Genome Browser (URL http://rohsdb.cmb.usc.edu/GBshape/cgi-bin/hgPcr, accessed on 15 March 2020) (Supplementary Table S1). Standard PCR amplification was performed using PCR Master Mix (Promega Corporation, Madison, WI, USA) with an initial denaturing step of 95 °C for 2 min, 38 cycles of 95 °C for 45 s, 55 °C for 45 s, and 72 °C for 45 s, and a final elongation at 72 °C for 5 min. PCR products were purified using ExoStar1 Step (Euroclone S.p.A, Pero, Italy), directly sequenced on both strands using Big DyeTerminator V3.1 (ThermoFisher Scientific, Waltham, MA, USA), and subsequently resolved on an ABI3500 Genetic Analyzer (Applied Biosystems, Foster City, CA, USA). For the identification of heterozygous variants, the electropherogram of Sanger sequencing was observed, while the presence of homozygous variants was investigated by sequence alignment with the GRCh38/hg38 human genome assembly using the BLAT function of the UCSC Genome Browser. All analyses, in order to exclude preanalytical and analytical errors, were repeated on PCR products obtained from new nucleic acid extractions.

### 2.3. Periodontal Pathogens

The results of the bacterial load based on real-time PCR of the patient sample, expressed as copy number/mL, are shown in Table 1. The highest value was observed for *Tannerella Forsythia*, *Porphyromonas Gingivalis*, and *Aggregatibacter Actinomycetemcomitans* with 6.200.000, 16.000, and 13.400 copies/mL, respectively. Lower values were present for *Prevotella Intermedia*, *Porphyromonas Endodontalis*, and *Treponema Denticola* with 7.900, 3.700, and 2.100 copies/mL, respectively. The least represented bacterium was *Fusobacter Nucleatum* with 1.740 copies/mL.

The results of the mutational analysis carried out for the VDR polymorphisms and for the IL-1α, IL-1β, IL6, and IL10 polymorphisms are summarized in Table 2. Concerning the VDR polymorphisms, the results indicated FokI with a homozygous (mutated) genotype (TT-ff), while the SNPs BsmI, ApaI, and TaqI were all in heterozygosity. Sequence analysis also revealed homozygosity for IL1B rs1143634 (CC), IL10 rs1800896 (AA), and rs1800871 (TT), whereas IL1A rs1800587 (CT) and IL6 rs1800795 (GC) were heterozygous.

**Figure 4:**
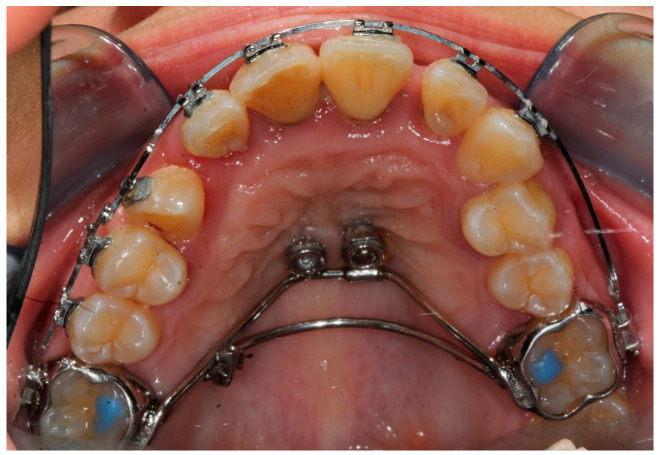
Orthodontic appliance: the device is anchored on molar bands on the upper first molar and the palatal screw. This device was used to pull the impacted canine into place in the dental arch.

From the results of the genetic analysis of interleukins, the patient was judged to be at risk of root resorption. Furthermore, the results of the genetic analysis on the bacterial load suggested prudence and attention in checking the periodontium of the teeth on which the orthodontic appliance would be applied, in addition to planning the periodontal therapy. For this reason, in the orthodontic therapy project, we reduced the forces on the anchoring teeth of the appliance. A device anchored on molar bands on the upper first molars and two palatal screws (Dentaurum, Ispringen, Germany) was constructed, the positioning of which occurred using a surgical guide (Figure 4).

**Figure 5:**
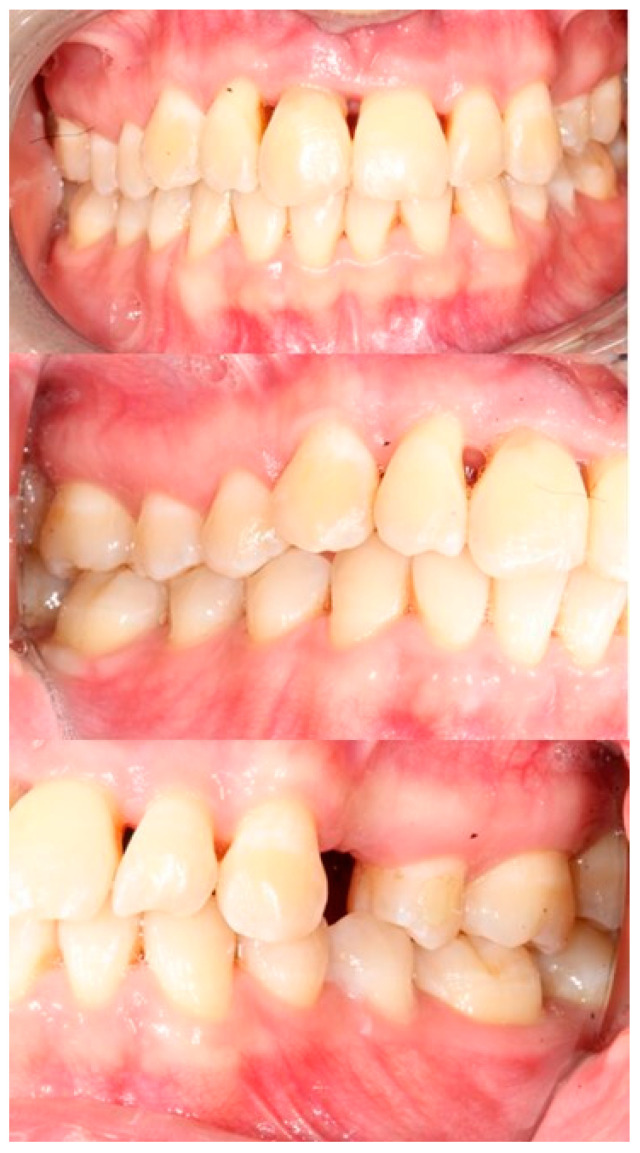
Intraoral photograph at the end of orthodontic treatment.

The use of this hybrid anchoring system, dental and on screws, allows to reduce the forces exerted on the supporting teeth. The device had a palatal arm that could be activated with an eyelet to be able to tie the elastic traction. The day after screw insertion, the right screw showed mobility and was removed. The stability of the device was considered valid, therefore, the screw was not replaced. The impacted canine was surgically exposed, and a button was bonded to the crown with a chain for traction under cover. The orthodontic treatment lasted from September 2019 to December 2022. When the impacted canine appeared in the palate, in March 2021, the orthodontic brackets were bonded to the teeth of the upper arch, except for the two left premolars in good occlusion. In the right sector of the anterior area, it was necessary to improve the alignment and increase the space for the right canine. The orthodontic brackets were applied from canine to canine and on the right premolars to distribute the orthodontic forces, always applied with the lowest possible intensity. The aim was to improve alignment, resolve the cross bite of the left lateral incisor, and position the right canine along the dental arch. For the therapy, round section wires were used, starting with preformed Nickel Titanium .012″ archwires and then progressively .014″ and .016″, ending with a .016″x.022″ stainless steel archwire only after aligning teeth. The archwires were tied to the brackets using short .010″ metal ligatures, tightened progressively until the desired tooth movement was completed. In October 2022, during the last stages of orthodontic therapy, the patient reported pain and mobility of the upper left first premolar not included in the orthodontic appliance. An incomplete tooth fracture, a mesio-distal crack that extended occlusogingivally into the root was diagnosed by endodontist using a dental operating microscope (LABOMED, Los Angeles, CA, USA). Since the patient was seen once a month, the periodontal damage occurred quickly, and the tooth had to be extracted [31]. The first left premolar was not included in the orthodontic appliance, but it had a good relationship with the antagonist and had never shown clinical signs during the treatment period. The speed of bone destruction can be traced back to the patient’s characteristics, highlighted by genetic analyses. Unfortunately, in these cases, the most suitable therapy is the extraction of the fractured tooth. The patient completed orthodontic treatment and was referred to the periodontist to maintain her periodontal health, which was at risk due to her diabetes (Figure 5).

The patient, although receiving personalized periodontal supportive therapy concomitant during orthodontic therapy, presented, in the late stages of treatment, worsening of the periodontal condition, especially in the area of the upper incisors, with the presence of a gingival recession.

## 3. Discussion

ECR is a form of external tooth resorption. It is a pathological and aggressive phenomenon that usually leads to a significant and progressive loss of dental structure [32]. ECR usually starts coronally to the junctional epithelium [23,33] at the cervical part of the root and has a three-dimensional conformation expanding vertically and horizontally toward the root canal. The resorptive process and highly vascular resorptive tissue can produce a typical pinkish discoloration of the enamel, an irregular gingival contour, and a cervical cavitation [34]. However, the affected teeth are usually asymptomatic in the early stage, and the diagnosis is made using incidental radiographic findings [32]. Periapical radiographs are not considered sufficiently accurate in understanding the ECR phenomenon, and many authors have shown that cone beam computed tomography (CBCT) is the most accurate method for detecting ECR [35,36]. A clinical classification has been developed by Heithersay, dividing ECR into four bi-dimensional classes: class 1 and class 2 denote respectively a small and a well-defined invasive resorptive lesion, whereas class 3 and 4 respectively denote a deeper and a large invasive resorptive lesion extending into the coronal third of the root [37]. In 2017, Patel et al. proposed a new, more complex 3D classification thanks to the evaluation of the ECG by using CBCT [34].

The etiology of ECR is uncertain, but different potential predisposing factors have been associated with it [37]. History of trauma and orthodontic treatment are reported to be the most frequent risk factors [37,38]. Furthermore, a history of intra-coronal bleaching, dentoalveolar surgery, and periodontal treatment have been identified as other predisposing factors. Finally, a potential genetic predisposition has also been recognized, especially in multiple cervical root resorption (MCRR) [27,39].

ECR treatment aims to remove resorptive tissue, fill the defect with biocompatible and aesthetic materials, and avoid ECR recurrence [32]. ECR treatment can involve an external or internal approach with or without root canal treatment. When the defect of resorptive cases cannot be reached surgically, for example, the interproximal or middle/apical-third of roots, intentional reimplantation can be evaluated [28].

Regarding crack tooth syndrome, teeth with intra-coronal restorations are reported to have a greater incidence and prevalence of cracks [40] even if other studies have reported an increasing incidence in unrestored teeth [31,41]. As in this case report, many authors have showns that the most prevalent crack occurs in the mesio-distal direction [40,42,43] and most of all, they are found in posterior teeth, particularly the mandibular molars, maxillary molars, and maxillary first premolars [44,45]. The suspicion is that this crack occurs due to excessive masticatory force even if many causes have been described: thermocycling fatigue [46], eating coarser foods, the habit of biting on hard objects like opening beer bottle caps with one’s teeth, or [47] parafunctional habits like bruxism [47]. The isolated probing pocket depth was more than 10 mm in the line of the crack caused also by the periodontal status of the patients. Periodontal conditions and mesio-distal cracks lead to tooth extraction [48].

The abundant presence of pathogenic bacteria found in the patient is based on her genetic condition of type 1 diabetes and the presence of pathogenetic variants of VDR. Concerning VDR, there is a paucity of data in the literature describing a correlation between periodontopathogenic bacterial species and predisposing genetic factors, such as polymorphisms, with the disease. Moreover, the use of these diagnostic and prognostic parameters, which are indicative of personalized medicine, is far from routine in current clinical practice.

We analyzed the genetic variants of VDR because it is now recognized that these polymorphisms play a certain role in the susceptibility to periodontal disease. Furthermore, increasing evidence suggests a potential association with the presence and levels of periodontopathogenic bacteria. Our data showed a homozygous state for FokI (TT-ff), while the remaining BsmI, ApaI, and TaqI polymorphisms were all in the heterozygous state.

As reported in the literature and as found and confirmed in our previous study, there is a correlation between the homozygous T/T (f/f) genotype of the FokI variant, which results in the production of lower protein levels, and high bacterial load [9,49]. It has also been reported that the T allele (f) is associated with lower bone mineral density [50] and significantly higher susceptibility to infection and sepsis [51].

Regarding the other polymorphisms in heterozygosity, we should mention that the G allele (b) for BsmI is associated with low bone density [52] and the T allele (T) for TaqI increases the risk of developing periodontitis [50].

In the patient who came to our attention, among the results obtained from the evaluation of the bacterial load, the first thing we noticed was the presence of a high level of the ‘red complex’, including *Tannerella forsythia*, *Treponema denticola*, and *Porphyromonas gingivalis*. These are strongly associated with oral diseases; between them, there is a synergistic interaction and metabolic interdependence [53].

Diabetes is one of the most important risk factors for periodontitis. The new classification of periodontal diseases introduced in 2017 defines diabetes as a grade modifier of the progression of periodontal disease. A patient who presents levels of HbA1c ≥7.0 shifts to a rapid grade of progression of periodontal disease despite other risk factors [54]. Diabetes and periodontal disease are related by a two-way relationship. The most accepted hypothesis postulates that diabetes mellitus could boost the inflammatory response of periodontal tissues, upregulating the production of pro-inflammatory cytokines and MMPs and altering Polymorphonuclear neutrophil (PMN) function. On the other hand, Advanced Glycation End-Products (AGE) accumulate in the periodontal tissues of diabetics. They are responsible for the overproduction of inflammatory mediators such as IL-1b, TNFα, and IL-6. AGEs also have a harmful effect on bone metabolism, inducing alteration of bone formation and repair mechanisms [55,56].

Concerning the changes that diabetes may cause in the composition of subgingival microbiota, there are relatively few studies. In general, they suggest that there are more similarities than differences between diabetic and non-diabetic subjects. Nevertheless, some significant differences have been detected, such as the greater prevalence of *Porphyromonas gingivalis* and *Prevotella intermedia* in diabetic subjects [57].

Interleukin polymorphisms have also been extensively studied in patients with periodontal disease for their role in host genetic predisposition to dysregulated immune responses [7,58]. The results of this study first highlight the homozygous status of SNPs in IL10. IL10 exerts a powerful anti-inflammatory action by causing T-cell anergy and reducing the production of various pro-inflammatory cytokines. Among various polymorphisms in the IL10 gene promoter region, SNPs in homozygosity rs1800896 AA and rs1800871 TT are associated with reduced production of serum IL10 [59]. Lack of IL10 expression correlates with a high susceptibility to alveolar bone loss in knockout mice [60,61] and it is now well established that reduced expression, with decreased anti-inflammatory and increased pro-inflammatory mediators, can lead to bone loss-related diseases such as osteoporosis and periodontitis [59,62,63,64].

The IL1A and IL1B genes are located close to each other on the q-arm of chromosome 2 and have common DNA sequences [65]. Our results showed IL1B rs1143634 (CC) and IL1A rs1800587 (CT) as being homo- and heterozygous, respectively. The IL1A rs1800587 and IL1B rs1143634 polymorphisms are both characterized by the substitution of the cytosine nucleotide by thymine, and several studies have shown that the presence of the minor T allele, as in our case only for rs1800587, significantly increases IL1α and IL1β production in vitro respectively [58]. Furthermore, data from previous studies indicate that the composite genotype of the two polymorphisms, with the presence of the T allele in both, correlate with the incidence and severity of periodontal inflammation in numerous experimental studies and meta-analyses [5,65]. In this regard, there are promising results concerning probiotic therapy in oral health and a great stimulus to research in the area to demonstrate the probiotic strains with improved capacity and product concentrations and vehicles [66]. Probiotic bacteria may reverse damage to epithelia, caused by inflammation, by stimulating the upregulation of structural proteins. These bacteria may also colonize and proliferate sufficiently to deprive pathogenic bacteria of nutrients and thus inhibit their growth. Probiotic bacteria have also been reported to produce antimicrobial products, such as acetic acid and lactic acid, that inhibit Gram-negative bacteria. Moreover, probiotics may influence the host to downregulate pathways that might damage host tissues, while simultaneously upregulating other pathways that inhibit the growth or virulence of pathogens [67].

The SNP rs1800795 of IL6, identified in our study as being heterozygous (GC), located in the proximal promoter of the gene, can modulate the binding affinity of various transcription factors, and the C allele has been shown to be associated with a lower serum level of IL6 than the G allele [58,68]. A large body of experimental evidence suggests that the presence of the G allele, and thus the increased expression of IL6, is associated with periodontal disease, inducing osteoclast differentiation and bone resorption and inhibiting bone formation [58,69].

The literature suggests not applying intense force to avoid root resorption [70]. In the clinical case presented, orthodontic therapy was carried out by minimizing the intensity of the force. During the extrusion of the impacted canine, the forces on the anchoring teeth were reduced by using a palatal miniscrew. During the alignment phase, all measures were used to reduce the force applied to the teeth.

In conclusion, this complex clinical case shows how important it is to establish the right treatment plan with a multidisciplinary approach involving several specialists in the field of dentistry. Furthermore, in the future, genetic analysis could become an important step in establishing the most suitable therapy and helping in research on different dental diseases.

## Figures and Tables

**Table 1:** Real-time PCR bacterial load results from the patient in this study.

Periodontal Pathogens	Patient in This Study Copies/mL
Tannerella Forsythia	6.200.000
Porphyromonas Gingivalis	16.000
Aggregatibacter Actinomycetemcomitans	13.400
Prevotella Intermedia	7.900
Porphyromonas Endodontalis	3.700
Treponema Denticola	2.100
Fusobacter Nucleatum	1.740

**Table 2:** Identified genotypes of the VDR (FokI, BsmI, ApaI, and TaqI) and IL1A, IL1B, IL16, and IL10 SNPs. * Correspondence of nomenclature of SNP alleles in brackets.

Gene Variant	SNP ID	Nucleotide Change *	Genotype Identified
VDR FokI	rs2228570	c.2T > C (f > F)	TT (ff)
VDR BsmI	rs1544410	c.1024 + 283G > A(b > B)	GA (bB)
VDR ApaI	rs7975232	c.1025 −49A > C(A > a)	AC (Aa)
VDR TaqI	rs731236	c.1056T > C (T > t)	TC (Tt)
IL-1α	rs1800587	c.-949C > T	CT
IL-1β	rs1143634	c.3954C > T	CC
IL-6	rs1800795	c.-174G > C	GC
IL-10	rs1800896	c.−1082 A > G	AA
IL-10	rs1800871	c.-819 T > C	TT

## Data Availability

Data are contained within the article.

## Supplementary Materials

The following supporting information can be downloaded at: https://www.mdpi.com/article/10.3390/bioengineering11101023/s1, Table S1. Primers set designed for PCR and sequence analysis for the IL1A (rs1800587), IL1B (rs1143634), IL-6 (rs1800795) and IL10 (rs1800896 and rs1800871) polymorphisms.
