# Supplementary material for: Genetic Testing as a Source of Information Driving Diagnosis and Therapeutic Plan in a Multidisciplinary Case
Source: Bioengineering (Basel). 2024 Oct 14;11(10):1023. doi: 10.3390/bioengineering11101023 (PMC11505315; doi:10.3390/bioengineering11101023)
Supplement: Supplementary file 1 [file bioengineering-11-01023-s001.zip › bioengineering-3241202-supplementary.pdf]

Table S1: Primers set designed for PCR and sequence analysis for the IL1A (rs1800587), IL1B (rs1143634), IL-6 (rs1800795) and IL10 (rs1800896 and rs1800871) polymorphisms.

Ref. SNP	Primer name	Sequence primer (5′ to 3′)	Sizes (bp)	T° annealing
rs1800587, c.-949	IL1A-F	CATGGCTTAAACTCCAACTGG	284bp	55°
	IL1A-R	TAGGATATGCCCAAGGTGTG		
rs1143634, c.3954	IL1B-F	AGGTCCAGTGTTCTTAGCCA	393bp	55°
	IL1B-R	GGAGAATTAGCAAGCTGCCA		
rs1800795, c.-174	IL6-F	CAAGACATGCCAAAGTGCTG	414bp	55°
	IL6-R	AGTTCCAGGGCTAAGGATTTC		
rs1800896, c.-1082	IL10-F	AGGTCAGTGTTCCTCCCAGT	524bp	55°
	IL10-R	AAGGGTACACCAGTGCCAAC		
rs1800871, c.-819	IL10-F2	TTCTCAGTTGGCACTGGTGT	519bp	55°
	IL10-R2	TCTCATTCGCGTGTTCCTAG		
